# Loss-of-function and missense variants in *NSD2* cause decreased methylation activity and are associated with a distinct developmental phenotype

**DOI:** 10.1038/s41436-021-01158-1

**Published:** 2021-05-03

**Authors:** Paolo Zanoni, Katharina Steindl, Deepanwita Sengupta, Pascal Joset, Angela Bahr, Heinrich Sticht, Mariarosaria Lang-Muritano, Conny M. A. van Ravenswaaij-Arts, Marwan Shinawi, Marisa Andrews, Tania Attie-Bitach, Isabelle Maystadt, Newell Belnap, Valerie Benoit, Geoffroy Delplancq, Bert B. A. de Vries, Sarah Grotto, Didier Lacombe, Austin Larson, Jeroen Mourmans, Katrin Õunap, Giulia Petrilli, Rolph Pfundt, Keri Ramsey, Lot Snijders Blok, Vassilis Tsatsaris, Antonio Vitobello, Laurence Faivre, Patricia G. Wheeler, Marijke R. Wevers, Monica Wojcik, Markus Zweier, Or Gozani, Anita Rauch

**Affiliations:** 1 Institute of Medical Genetics, University of Zürich, Schlieren-Zurich, Switzerland; 2 Department of Biology, Stanford University, Stanford, CA USA; 3 Institute of Biochemistry, Friedrich-Alexander University Erlangen-Nürnberg, Erlangen, Germany; 4 Department of Pediatric Endocrinology and Diabetology, University Children’s Hospital, Zurich, Switzerland; 5 Children’s Research Centre, University Children’s Hospital, Zurich, Switzerland; 6 Department of Genetics, University of Groningen, University Medical Centre Groningen, Groningen, The Netherlands; 7 Department of Pediatrics, Division of Genetics and Genomic Medicine, Washington University School of Medicine, St. Louis, MO USA; 8 Service d’Histologie-Embryologie-Cytogénétique, Unité d’Embryofoetopathologie, Hôpital Necker-Enfants Malades, APHP, Paris, France; 9 INSERM UMR 1163, Université de Paris, Imagine Institute, Paris, France; 10 Centre de Génétique Humaine, Institut de Pathologie et de Génétique, Gosselies, Belgium; 11 Faculté de médecine, Université de Namur, Namur, Belgium; 12 Center for Rare Childhood Disorders (C4RCD), Translational Genomics Research Institute, Phoenix, AZ USA; 13 Neurogenomics Division, Translational Genomics Research Institute, Phoenix, AZ USA; 14 Département de Biologie Moléculaire, Institut de Pathologie et de Génétique, Gosselies, Belgium; 15 Centre de Génétique Humaine, Université de Franche-Comté, CHU, Besançon, France; 16 Service de Neuropédiatrie, CHU, Besançon, France; 17 Department of Human Genetics, Radboud University Medical Center, Nijmegen, The Netherlands; 18 Maternité Port-Royal, AP-HP, Hôpital Cochin, Paris, France; 19 Service de Génétique Médicale, Hôpital Pellegrin CHU, Bordeaux, France; 20 Department of Pediatrics, Section of Genetics, University of Colorado Anschutz Medical Campus, Denver, CO USA; 21 Deventer Ziekenhuis, Deventer, the Netherlands; 22 Department of Clinical Genetics, United Laboratories, Tartu University Hospital, Tartu, Estonia; 23 Department of Clinical Genetics, Institute of Clinical Medicine, University of Tartu, Tartu, Estonia; 24 UFR Des Sciences de Santé, INSERM-Université de Bourgogne UMR1231 GAD, FHU-TRANSLAD, Unité Fonctionnelle D’Innovation en Diagnostique Génomique Des Maladies Rares, Pôle de Biologie, CHU Dijon Bourgogne, Dijon, France; 25 Centre de Référence Maladies Rares « Anomalies du Développement Et Syndrome Malformatifs » de L’Est, Hôpital D’Enfants, FHU-TRANSLAD, CHU Dijon Bourgogne, Dijon, France; 26 Division of Genetics, Arnold Palmer Hospital, Orlando Health, Orlando, FL USA; 27 Department of Pediatrics, Boston Children’s Hospital, Divisions of Newborn Medicine and Genetics and Genomics, Boston, MA USA; 28 The Broad Institute of MIT and Harvard, Cambridge, MA USA; 29 Zurich Center for Integrative Human Physiology, University of Zurich, Zurich, Switzerland; 30 Neuroscience Center Zurich, University of Zurich and ETH Zurich, Zurich, Switzerland

## Abstract

**Purpose:**

Despite a few recent reports of patients harboring truncating variants in *NSD2*, a gene considered critical for the Wolf–Hirschhorn syndrome (WHS) phenotype, the clinical spectrum associated with *NSD2* pathogenic variants remains poorly understood.

**Methods:**

We collected a comprehensive series of 18 unpublished patients carrying heterozygous missense, elongating, or truncating *NSD2* variants; compared their clinical data to the typical WHS phenotype after pooling them with ten previously described patients; and assessed the underlying molecular mechanism by structural modeling and measuring methylation activity in vitro.

**Results:**

The core *NSD2*-associated phenotype includes mostly mild developmental delay, prenatal-onset growth retardation, low body mass index, and characteristic facial features distinct from WHS. Patients carrying missense variants were significantly taller and had more frequent behavioral/psychological issues compared with those harboring truncating variants. Structural in silico modeling suggested interference with NSD2’s folding and function for all missense variants in known structures. In vitro testing showed reduced methylation activity and failure to reconstitute H3K36me2 in *NSD2* knockout cells for most missense variants.

**Conclusion:**

*NSD2* loss-of-function variants lead to a distinct, rather mild phenotype partially overlapping with WHS. To avoid confusion for patients, *NSD2* deficiency may be named Rauch–Steindl syndrome after the delineators of this phenotype.

## INTRODUCTION

Wolf–Hirschhorn syndrome (WHS, also known as 4p-syndrome, OMIM 194190) caused by partial deletions of the short arm of chromosome 4 is one of the first microscopically recognized structural chromosomal disorders, and was named after Ulrich Wolf (Freiburg, Germany) and Kurt Hirschhorn (New York, NY, USA), who first described it in 1965.^1,2^ Hallmarks of the syndrome are prenatal-onset growth deficiency, microcephaly, intellectual disability (ID), epilepsy, muscular hypotonia and hypotrophy, facial clefts, congenital heart defects and other malformations, as well as a very characteristic craniofacial gestalt, often described as resembling a Greek helmet.^3^ With the advent of molecular cytogenetics a number of patients with smaller deletions encompassing 4p16.3 were identified, with deletions below 3.5 Mb found to be associated with a milder phenotype without major malformations.^3^ Following an observation by Dian Donnai (Manchester, UK)^4^ individuals described as having Pitt–Rogers–Danks syndrome were shown to also harbor 4p16.3 microdeletions sizing at least 3.7 Mb^5^ and, as pointed out by Agatino Battaglia (Pisa, Italy) and John C. Carey (Salt Lake City, UT, USA), are actually clinically indistinguishable from WHS.^6^ Further refinement of the WHS critical region hinted at *NSD2* (also named *WHSC1* or *MMSET*, OMIM *602952) as the gene possibly responsible for part of the manifestations of WHS, including growth retardation, ID, and possibly susceptibility to infections.^7–10^ This hypothesis was supported by a role for *NSD2* in neuronal development as well as in the regulation of cellular proliferation in vitro.^11–13^ A total of eight truncating, two splicing, and six missense variants were listed among many others in sequencing studies of patient cohorts with neurodevelopmental disorders, growth abnormality, or congenital heart defects without deep phenotyping information (Table S1).

Recently, eight individual patients with developmental delay (DD) and growth retardation carrying truncating or protein-elongating variants in *NSD2* have been described,^14–18^ with phenotypes that partially overlap with those of individuals carrying very small chromosomal deletions encompassing *NSD2*.^8–10^

However, the lack of larger systematic patient series and functional validation is hampering the understanding of the phenotype associated with *NSD2* constitutional variants. Here we report 18 additional deep-phenotyped individuals carrying heterozygous (likely) pathogenic *NSD2* variants, including a prenatal and two familial cases, together with in silico and in vitro data providing insight into the underlying pathomechanism and genotype–phenotype correlations.

## MATERIALS AND METHODS

The 18 previously undescribed patients with pathogenic *NSD2* variants reported in this study were identified by research or diagnostic exome or Mendeliome sequencing in various laboratories and collected via GeneMatcher (see Web Resources). When no developmental testing was performed, the degree of ID was estimated using the *Diagnostic and Statistical Manual of Mental Disorders, Fifth Edition* (DSM-5) severity levels for ID (see Web Resources). Standard deviation scores for growth parameters were calculated based on the data sets provided by the Swiss Society of Pediatrics, which combine World Health Organization, Swiss, and German population data (see Web Resources). The facial overlay Fig. 4b was obtained using the Face2Gene RESEARCH application (FDNA Inc., Boston, MA, USA) taking one frontal photo for each patient at the youngest available age.

*NSD2* variant nomenclature refers to the NM_133330.2 transcript and pathogenicity classification is based on the American College of Medical Genetics and Genomics/Association for Molecular Pathology (ACMG/AMP) guidelines.^19^ Structural analysis of NSD2 variants was performed using SwissModel, RasMol, and Smart (see Web Resources). While the experimental crystal structure of the NSD2 N-methyltransferase domain was available (Protein Data Bank, PDB: 5LSU), the PHD and PWWP domains were modeled using the homologous domains from TRIM24 (PDB: 4ZQL) and NSD3 (PDB code: 2DAQ), respectively.

GST fusion proteins were obtained as described in the Supplementary methods. In vitro methylation assays were performed as described in Mazur et al.^20^ and the Supplemental methods using reagents listed in Table S2.

The data in Fig. 2n, p, r, t are represented as mean ± SD of two independent biological replicates; statistical significance was tested by one-way analysis of variance (ANOVA) followed by two-tailed Dunnett’s test without adjusting for multiple testing. The data in Fig. 3c–e are represented as mean ± SD. Groups in Fig. 3e were compared by homoscedastic two-tailed Student’s *t*-test after testing for equal variance by *F*-test.

The graphs in Figs. 2n, p, r, t and 3a–f were generated using GraphPad Prism version 5.00 for Windows, (GraphPad Software, La Jolla, CA, USA). All statistical analyses were performed with GraphPad Prism, except for *F*- and *t*-tests, which were performed using Microsoft© Excel.

## RESULTS

**Fig. 1:**
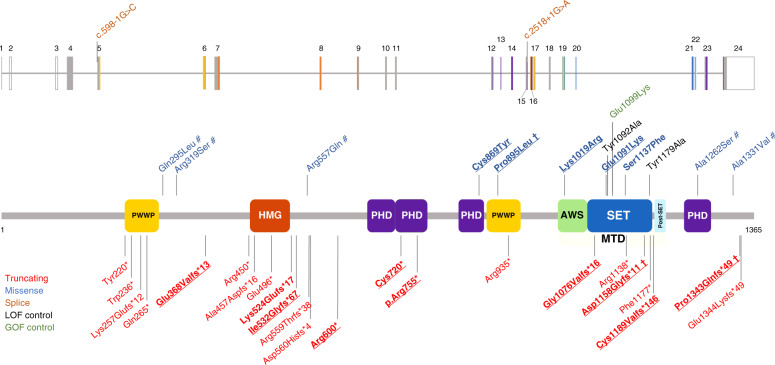
Localization of the *NSD2* variants. The diagram shows the structure of the *NSD2* gene (above) and protein (below, isoform 1, encoded by the NM_133330.2 transcript) together with the variants discussed in this study as well as variants that have previously been reported in large sequencing studies. The variants carried by the 18 additional individuals described in this study are in bold. Observed germline variants absent in the HGMD database are underlined. † Shared by more than one patient; # variant of uncertain significance (VUS)/likely benign. The observed pathogenic missense variants we found map to three distinct domains of NSD2: a PHD zinc-finger domain (residues 831–875), a PWWP domain (residues 880–942), and the catalytic methyltransferase domain (residues 1011–1203; composed of three subdomains, namely an AWS, a SET, and a post-SET domain). The color coding of the variants and protein domains is reported in the legend. AWS Associated With SET domain (IPR006560), GOF gain of function, HMG high mobility group box domain (IPR009071), LOF loss of function, MTD catalytic methyltransferase domain, composed by the AWS, SET and a post-SET domain, PHD zinc-finger domain, Plant-HomeoDomain type (IPR001965), PWWP proline–tryptophan–tryptophan–proline domain (IPR000313), SET Su(Var)3-9, enhancer-of-zeste, trithorax domain (IPR001214). Domains were annotated according to the Uniprot databank (see Web Resources).

We describe 18 patients, 16 of whom unrelated, carrying ultrarare (absent in gnomAD) pathogenic or likely pathogenic *NSD2* variants (Table S3). Of these, 6 carried 5 different missense variants while 12 carried 10 different truncating variants. Nine of the truncating and four of the missense variants were not reported previously (Fig. 1). Variant inheritance could not be determined in 4/18 cases, and was confirmed parental in 3/18 cases and de novo in 11/18 cases.

**Fig. 2:**
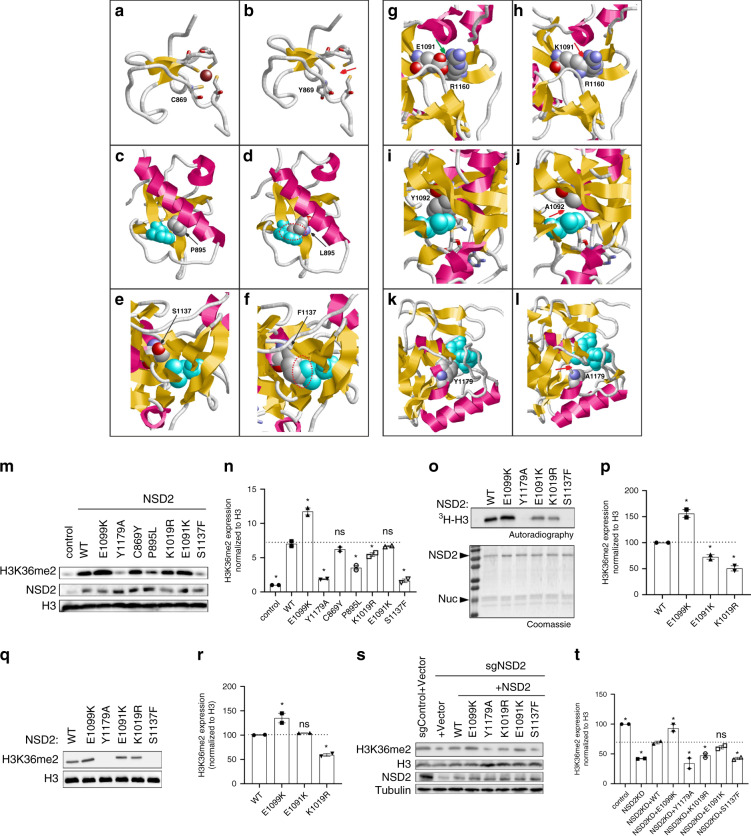
Structural and functional effect of two synthetic and four naturally occurring pathogenic *NSD2* missense variants. (**a**) Wild-type (WT) Cys869 is one of four cysteines that tetrahedrally coordinate a zinc ion (Cys846, Cys849, Cys869, Cys872 are shown in stick presentation; the Zn^2+^ is depicted as a brown ball). (**b**) The variant Tyr869 adopts a different sidechain orientation resulting in a loss of the zinc ion (marked by a red arrow). (**c**) WT Pro895 is located in spatial proximity of Trp885 (cyan). (**d**) The longer Leu895 sidechain present in the variant results in steric clashes with Trp885 (marked by a red dotted circle). (**e**) WT Ser1137 is located in spatial proximity of Leu1163 (cyan). (**f**) The bulkier Phe1173 sidechain present in the variant results in steric clashes with Leu1163 (marked by a red dotted circle). (**g**) WT Glu1091 forms a salt bridge to Arg1160 (green arrow), which is disrupted in the (**h**) Lys1091 variant and steric clashes are formed instead (red arrow). (**i**) WT Tyr1092 forms tight van der Waals interactions with Leu1120 (cyan), which are lost in the synthetic (**j**) Ala1092 variant^11^ (the site of altered interactions is denoted by a red arrow). (**k**) WT Tyr1179 forms stabilizing interactions to S-adenosylmethionine (SAM, cyan). (**l**) The shorter sidechain in the synthetic Ala1179 variant^11^ results in a loss of interactions with the SAM cofactor (red arrow), which is expected to result in a drastic loss of enzymatic activity. (**m**) Western analysis with the indicated antibodies of whole-cell extracts (WCEs) from 293 T cells overexpressing vector control, full-length WT NSD2, or NSD2 mutants as indicated. Histone H3 is shown as a loading control. (**n**) Quantification of western blot data in (**m**). (**o**) In vitro methylation assay with recombinant WT NSD2 or mutant NSD2 derivatives as indicated on recombinant nucleosomes (rNuc) as substrates. Top panel, ^3^H-SAM is the methyl donor and methylation is visualized by autoradiography and indicated as ^3^H-H3. Bottom panel, Coomassie stain of proteins in the reaction. (**p**) Quantification of all detectable bands in the autoradiography in (**o**). (**q**) Western analysis with the indicated antibodies of in vitro methylation assay with nonradiolabeled SAM. (**r**) Quantification of all detectable bands in the western blot data in (**q**). (**s**) Western analysis with the indicated antibodies of WCEs from WT or *NSD2* deficient HT1080 cells complemented with CRISPR-resistant *NSD2* (WT or mutants), or control as indicated. Histone H3 and tubulin are shown as loading controls. (**t**) Quantification of western blots in (**s**). The data in (**n**, **p**, **r**, **t**) are represented as mean ± SD of two independent experiments. *p < 0.05 based on a one-way analysis of variance (ANOVA) followed by two-tailed Dunnett’s test.

NSD2 is the principal enzyme that dimethylates histone H3 at lysine 36 (H3K36me2)^11^ in most cell types and tissues.^21^ H3K36me2 is an evolutionarily conserved histone modification linked to transcriptional activation. The (likely) pathogenic missense variants we found map to three distinct domains of NSD2. The Cys869Tyr (patient 1-I) substitution disrupts zinc binding within a PHD finger domain (residues 831-875) and hence induces improper folding and loss of function for this domain. Although the function of the NSD2 PHD domain is unknown, these domains are virtually always found on chromatin-associated proteins and may function as epigenetic reader domains (Fig. 2a, b). The Pro895Leu variant (patients 7-I and 7-II) is located in the core of one of NSD2’s PWWP domains (Fig. 2c), another motif that generally functions as an epigenetic reader.^22^ The leucine substitution at Pro895 is predicted to destabilize the domain through steric clashes with the adjacent Trp885 (Fig. 2d).

All remaining missense variants are located within the methyltransferase domain of NSD2. Lys1019Arg (patient 12-I) is located in a loop of the methyltransferase domain that exhibits high local mobility or is entirely missing in the isolated NSD2 crystal structure. This hampers reliable modeling but suggests that this region is flexible and might become stabilized upon protein–protein interactions, such as binding to the nucleosome substrate. Ser1137 is located in the domain’s core (Fig. 2e) and substitution to phenylalanine (p.Ser1137Phe, patient 10-I) is predicted to result in steric clashes with the adjacent Leu1163 residue (Fig. 2f), leading to domain destabilization. Glu1091 forms a salt bridge to Arg1160 in the wild-type structure (Fig. 2g), which is disrupted due to the charge inversion in the Glu1091Lys variant (patient 5-I, Fig. 2h). In addition, the longer Lys1091 sidechain forms steric clashes with Arg1160, which are expected to additionally destabilize the methyltransferase domain. To corroborate our in silico and in vitro data, we also tested previously functionally characterized substitutions within the methyltransferase domain, namely Glu1099Lys, Tyr1092Ala, and Tyr1179Ala. Glu1099Lys is a recurrent variant found somatically in multiple cancer types including childhood leukemia and enhances methylation activity to alter global histone methylation.^23^ Molecular modeling^23^ suggested that the positively charged lysine may disrupt the favorable histone–enzyme interactions leading to a gain of function. Tyr1092Ala and Tyr1179Ala were not previously identified in patients but had been shown to abrogate catalytic activity based on homology to other SET domain protein lysine methyltransferase enzymes.^11^ Tyr1092 forms tight hydrophobic interactions with Leu1120 in the protein core (Fig. 2I). Due to the shorter sidechain, these interactions are lost in the Ala1092 variant (Fig. 2j) thereby predicted to destabilize the protein domain. Modeling of the Tyr1179Ala variant reveals that Tyr1179 forms direct interactions with the bound methyl donor S-adenosylmethionine (SAM, Fig. 2k). These interactions are lost in the Tyr1179Ala mutant (Fig. 2l), resulting in a predicted strong reduction of cofactor binding and hence loss of enzymatic activity. Based on these data, we postulated that one functional consequence of the *NSD2* missense variants identified in patients with developmental disorders might be disruption of NSD2’s ability to generate H3K36me2.

To test this hypothesis, we determined H3K36me2 levels in HEK293T cells transiently transfected with *NSD2* wild-type or derivatives harboring the various variants (Fig. 2m, n). Consistent with previous work,^11,22^ overexpression of wild-type *NSD2* and a cancer-related *NSD2* gain-of-function mutant (Glu1099Lys [E1099K])^23^ caused a global increase in H3K36me2. Contrarily, the *NSD2* derivatives carrying variants observed in patients, apart from Cys869Tyr [C869Y] (patient 1-I) and Glu1091Lys [E1091K] (patient 5-I), significantly reduced the levels of H3K36me2 (Fig. 2m; quantitation in Fig. 2n). In particular, Ser1137Phe [S1137F] (patient 10-I) largely behaved like the negative control and the known *NSD2* catalytic mutant Tyr1179Ala [Y1179A] (Fig. 2m, n). Given that Ser1137Phe (patient 10-I) is located within the SET domain, the data suggest that this variant causes direct impairment of NSD2’s catalytic activity. To test this hypothesis, in vitro methylation assays were performed using a minimal domain of NSD2 that retains enzymatic activity^11^ and recombinant nucleosomes as substrates. As shown in Fig. 2o and q (quantitation in Fig. 2p and r, respectively), Ser1137Phe (patient 10-I) abrogates NSD2’s ability to methylate nucleosomes in vitro similarly to the previously characterized catalytic mutant Tyr1179Ala [Y1179A]), while Glu1091Lys (patient 5-I) and Lys1019Arg [K1019R] (patient 12-I) showed a modest reduction in methylation of nucleosomes (Fig. 2o–r). Cys869Tyr (patient 1-I) and Pro895Leu (patients 7-I and 7-II) lie outside of the minimal catalytic domain and thus could not be tested in this assay. Next, we used the CRISPR/Cas9 system to generate *NSD2*-depleted HT1080 cells, which leads to depletion of global H3K36me2, and then complemented these cells with CRISPR-resistant wild-type *NSD2* or the DD-associated *NSD2* variants to test their role in reconstituting physiologic levels of H3K36me2. In contrast to complementation with wild-type *NSD2* and gain-of function *NSD2*-Glu1099Lys, cells complemented with the DD-associated *NSD2* variants were partially to largely compromised in their ability to rescue physiologic H3K36me2 levels, with Glu1091Lys (patient 5-I) being the only variant that did not reach statistical significance (Fig. 2s, t). The Ser1137Phe variant (patient 10-I) completely failed to rescue, indicating that it essentially abolishes enzymatic activity. Overall, these data are consistent with all variants except Cys869Tyr (patient 1-I) compromising to varying degrees the ability of NSD2 to generate H3K36me2, a key epigenetic modification.

**Fig. 3:**
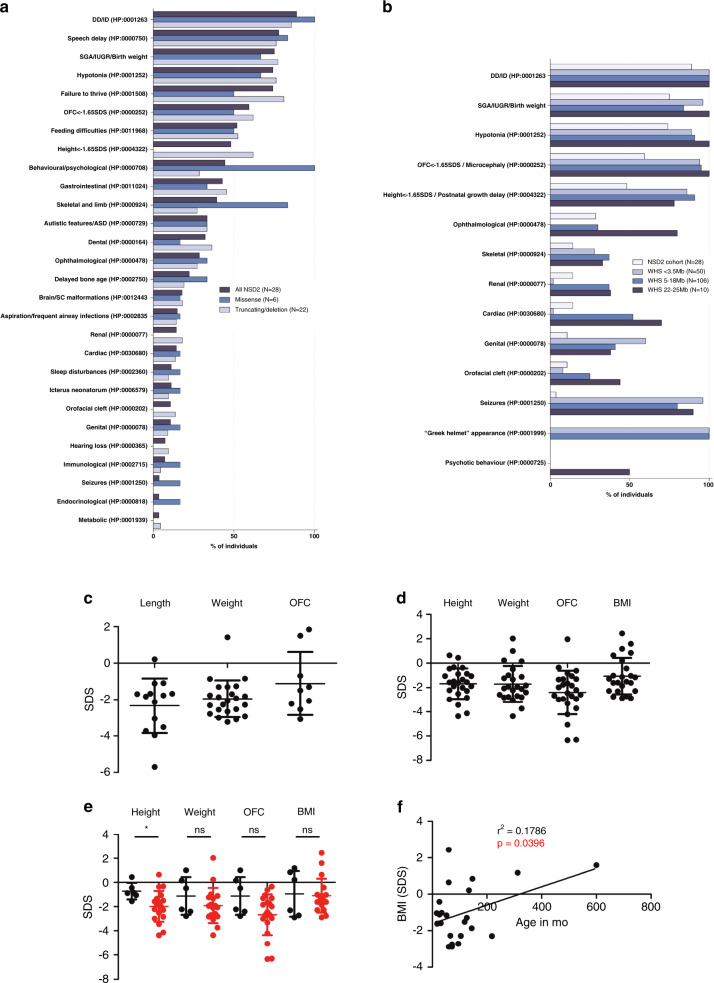
Phenotypic features and growth parameters of the individuals carrying *NSD2* pathogenic variants and comparison with Wolf–Hirschhorn syndrome (WHS). The diagram in (**a**) summarizes the phenotypic features of the individuals described in this study (18 additional patients together with 10 previously reported individuals) based on the type of *NSD2* variant carried. In (**b**) all individuals are compared with WHS patients, subdivided according to the size of the 4p deletion carried, as reported in Zollino et al.^3^ Data in (**a**) and (**b**) are expressed as percentages. Growth parameters at birth (**c**; included the parameters at termination of pregnancy for individual 16-I) and at last visit (**d**) are reported. The average measures for all combined individuals standard deviation score (SDS [SD]) were length (L): -2.3 (1.5); weight (W): -2.0(1.0); occipitofrontal circumference (OFC): -1.1(1.7); gestational weeks (GW): 38.5(3.8) at birth and height (H): -1.7(1.3); W: -1.7(1.5); OFC: -2.4(1.8); body mass index (BMI): -1(1.5) at last visit. (**e**) Comparison of the growth parameters at last visit for patients carrying missense variants (black) compared with patients carrying other variants as well as deletions encompassing *NSD2* (red). *p value < 0.05 as tested by two-tailed Student’s *t*-test. The data in (**c**–**e**) are expressed as SDS based on the respective growth charts. Lines and whiskers represent mean ± SD. (**f**) Linear correlation analysis between age and BMI and last visit. ASD DD/ID developmental delay/intellectual disability, IUGR intrauterine growth restriction, SC spinal cord, SGA small for gestational age.

The main clinical features of the *NSD2* cohort consisting of 18 individuals described here and 10 previously published individuals (8 carrying truncating or protein-elongating variants^14–18^ and 2 carrying a microdeletion that encompasses the *NSD2* gene only^10^) are summarized in Fig. 3a and compared to patients with WHS due to 4p deletions of different sizes reported in a large series of 166 affected individuals^3^ (Fig. 3b). Postmortem pathological findings for case 16-I, a male fetus whose pregnancy was terminated at the 27th week of gestation, are shown in Figure S1. Detailed clinical descriptions are provided in the supplement.

In the *NSD2* cohort, the average age at last visit (±SD) was 9.3 (±10.3) years and the male to female ratio was 18:10 (male 64%, female 36%). Seventy-five percent of the *NSD2* variants were de novo, 14% were inherited from an affected parent, while in 11% of the cases, the inheritance pattern could not be established.

**Fig. 4:**
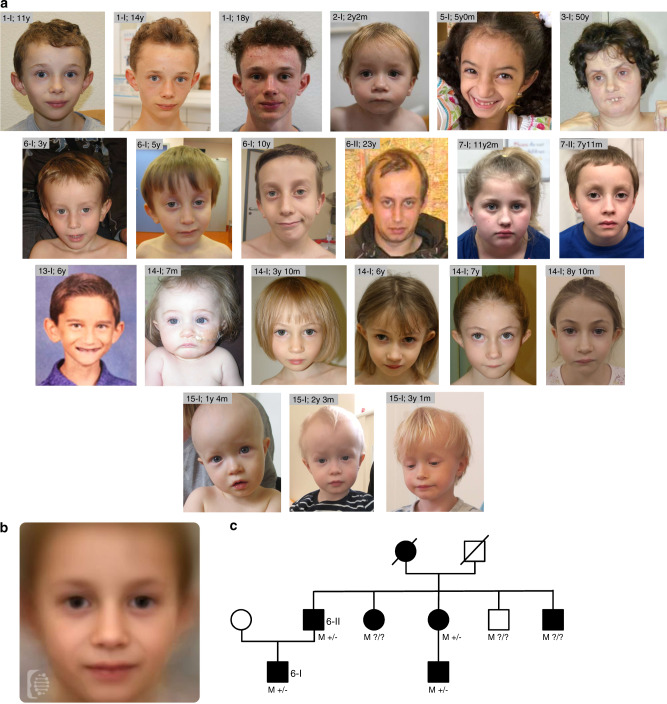
Clinical data. (**a**) Facial features of the affected individuals reported in this study. The age in years (y) and months (m) is reported beside each patient ID. (**b**) Combined facial gestalt obtained by combining frontal photos using the Face2Gene online research tool (see Materials and Methods and Web Resources). Photos from 12 different patients, including those depicted in (**a**), were used for this purpose. (**c**) Family tree of family number 6 in this study. M+/−/? = presence (+), absence (−), or unknown status (?) for the p.Asp1158Glyfs*11 variant in each *NSD2* allele. Clinically affected individuals are shown as full symbols.

Patients with (likely) pathogenic *NSD2* variants shared a similar facial gestalt characterized by a triangular face, broad forehead, high anterior hairline, deeply set eyes, large palpebral fissures, broad arched and laterally sparse eyebrows, periorbital hyperpigmentation, full cheeks, a thin and elevated nasal bridge, smooth short philtrum, prominent cupid bow, thick everted lower lip vermilion, and/or protruding ears (Fig. 4a, b). Noticeably, the facial appearance evolved over time, with older patients developing deeper infraorbital creases. Family 6 comprised a total of 7 clinically similarly affected individuals, with molecular confirmation available for four of them, and represents to date the largest known family affected by this disorder (Fig. 4c). The core phenotype of the *NSD2* cohort (>50% of patients) is characterized by DD, intrauterine growth retardation and low birth weight, feeding difficulties, failure to thrive, height and head circumference below the 5th centile (<−1.65 SDS), speech delay, and muscular hypotonia. Although these manifestations are present in WHS as well, the majority of the individuals in the *NSD2* cohort lack many of the most common manifestations of WHS such as seizures,^3^ orofacial clefts, and coloboma, as well as genital, cardiac, and renal malformations and display a distinct facial phenotype that lacks the classical “Greek helmet” aspect. Furthermore, the severity of the ID in the *NSD2* cohort was milder compared with patients carrying the most common 4p deletions (between 5 and 18 Mb), who are severely affected in 76% of the cases.^3^ While individuals 1-I, 13-I, and 14-I harboring the *NSD2* Cys869Tyr, p.Cys1183Valfs*146, and p.Arg600* variants respectively, either had an IQ in the lower-normal range (78–81 and 89, respectively) or presented with learning difficulties, individuals old enough for evaluation commonly showed mild ID, with only three individuals presenting with severe ID. The latter carried truncating variants (3-I c.3223_3226dup p.Gly1076Valfs*16; 4-1c.1588_1589dupAA p.Ile532Glyfs*67; Bernardini et al., patient 3, exon 1–20 deletion), which may not be held responsible for the severe ID. Both siblings described as patients 2 and 3 in Bernardini et al. carry in fact the same exon 1–20 deletion, with one sibling showing mild ID, while the other had autism and severe ID.^10^

Behavioral and psychological issues as well as autistic features were observed in 44% and 33%, respectively, with the most frequently reported manifestations being anxiety (15%), hyperactivity (22%), and aggressiveness (11%). Individual 1-I had suicidal ideations related to his poor academic performance at the age of 11 years. Of note, all the individuals carrying missense variants presented with behavioral and psychological issues (Fig. 3a), compared to 29% of patients with truncating variants or deletions (*p* value = 0.0031 by two-tailed Fisher’s exact test).

Growth parameters at birth were largely below the norm (Fig. 3c). Feeding difficulties were described in 52% of the affected individuals and may at least in part contribute to the failure to thrive. Short stature and growth retardation persisted later in life (Fig. 3d). Delayed bone age was detected in 6 individuals (22%). Length and occipitofrontal circumference (OFC) at birth as well as all growth parameters at last visit were normally distributed according to a D’Agostino–Pearson omnibus normality test, while birth weight was skewed toward lower values (*p* = 0.0002). Notably, heterozygotes for missense variants were significantly taller than patients carrying truncating variants as well as small deletions encompassing *NSD2* (*p* = 0.03 by homoscedastic *t*-test after checking for comparable variance by *F*-test; Fig. 3e) and none of them fell below the 5th centile for height at last visit. Weight and OFC at last visit were also higher in patients carrying missense variants, although this difference was not statistically significant (*p* = 0.27 and 0.17, respectively). Finally, a linear regression analysis showed that older patients had significantly higher body mass index (BMI) values (*r*² = 0.1786, *p* = 0.0396; Fig. 3f).

Gastrointestinal abnormalities were also quite common (43%), with constipation representing the most frequent manifestation (26%). Ophthalmological abnormalities were observed in 29% of patients and mostly included mild refraction defects and strabismus, while no individual presented with coloboma, which is considered a recurrent feature in WHS (see Fig. 3b). One exception is represented by individual 3-I, who presented with bilateral keratoconus, retinitis pigmentosa, and optic atrophy and received a corneal transplant at the age of 32. Reanalysis of her exome data revealed three rare variants of unknown significance in *RHO*, *KRT3*, and *UNC45B* (Table S3), which were, however, maternally inherited and also present in her healthy sister. Skeletal and limb abnormalities were reported in 39% of the cases. Individual 12-I presented with a craniosynostosis that was surgically corrected at the age of 6 months, which may be explained by the patient’s additional de novo heterozygous *AGO2* variant (Table S3). Also, of note, individual 7-II presented with 11 ossified ribs and 6 non-rib-bearing lumbar vertebrae. Dental abnormalities were also quite frequent (32%). Brain and spinal cord malformations were present in 5 individuals (18%) and were mostly of minor importance except for individual 16-I, who presented with vermis hypoplasia. Less frequent manifestations among patients carrying *NSD2* pathogenic variants included a history of aspiration, cardiac and renal anomalies, neonatal jaundice, sleep disturbances, hearing loss, genital abnormalities, and orofacial clefts (Table S3). Immunological abnormalities as well as recurrent infections, which represent a common morbidity and mortality cause in WHS,^24^ were infrequent in the *NSD2* cohort, where patient 5-I presented with latex allergy, patient 11-I with low IgA and IgG_3_ levels, and patients 2-I, 11-I and Derar-1 with recurrent respiratory infections. Seizures, which are common in WHS (Fig. 3b), were reported only in individual 10-I, while individual 15-I presented subclinical electroencephalogram **(**EEG) abnormalities at the age of 4 4/12 years. Endocrinological abnormalities seem not to be part of the phenotypic spectrum, with only individual 5-I presenting limited signs of precocious puberty at 15 months of age, which were nonprogressive and were associated with a normal endocrine evaluation. Likewise, metabolic abnormalities were only observed in individual 2-I.

## DISCUSSION

In this work we describe a large series of patients carrying pathogenic variants in *NSD2*, thus allowing delineation of the associated manifestations, which include prenatal-onset failure to thrive with all body measurements below the mean and low BMI, mild DD and muscular hypotonia, and a distinct facial gestalt. Furthermore, we provide in silico and in vitro mechanistic data showing loss of histone methylation activity as a common feature of *NSD2* deficiency.

The phenotype observed in the *NSD2* cohort is consistent with many of the known functions of *NSD2*. First, *NSD2* has long been known to regulate embryonic development and body growth, with heterozygous Nsd2 constitutive knockout mice growing at a much slower rate compared with wild-type littermates, homozygous knockout mice dying in the first days of life,^25^ and common variants showing strong (*p* = 10^−24^) association with height in genome-wide association study (GWAS).^26^ Recently *NSD2* was shown to regulate adipose tissue development in mice by controlling the activity of the master adipogenic transcription factor peroxisome proliferator-activated receptor-γ (PPARγ).^27^ Interestingly, in this work mice overexpressing the mutant histone protein H3.3K36M, an NSD2 inhibitor, resisted white adipose tissue expansion in response to a high-fat diet. These findings support the idea that the low BMI observed in the *NSD2* cohort may be due to the inability of body fat to expand in response to feeding rather than a consequence of the neonatal feeding difficulties that, although observed in multiple affected individuals, disappear later in life. Furthermore, *NSD2* has been shown to play a key role in promoting adipogenesis and myogenesis in precursor cells, as well as thermogenesis in brown adipose tissue and insulin sensitivity in white adipose tissue.^27^ In pancreatic β-cell lines in vitro *NSD2* has also been shown to promote proliferation and to regulate insulin secretion.^28^ However, since diabetes has not been reported as a recurrent feature neither in our cohort nor in patients with WHS we assume that in humans, glycemic control may be maintained despite impaired *NSD2* function.

Likewise, some other known or supposed functions of *NSD2* did not result in a recurrent phenotype in our cohort. *NSD2* has been suggested to contribute to the immune defects typical of WHS by regulating the hematopoietic process at multiple stages as well as B-^29^ and T-cell^30^ differentiation. Nevertheless, only a minority of the patients in this series presented with recurrent infections. Also, while heterozygous *Nsd2* deficient mice present with heart defects^25^ and de novo variants in *NSD2* were shown to associate with congenital heart malformations,^31^ cardiac anomalies were present only in a minority of the individuals in this series, and, with the exception of individual 16-I, were of minor clinical importance. The low prevalence of epilepsy in this cohort is compatible with the assumption that other genes such as *LETM1* are responsible for seizures in WHS.^32,33^

While heterozygotes for missense variants were on average significantly taller, the overall clinical severity correlated only loosely with the measured alteration in NSD2 enzymatic function. Furthermore, for the Cys869Tyr (patient 1-I) variant, which can be categorized as likely pathogenic based on the current ACMG/AMP recommendations,^19^ we did not detect significant loss of methylation activity in our in vitro assays. These data suggest that in addition to H3K36 dimethylation, other functions of NSD2 such as its genomic localization, and likely other potential genetic differences among the patients, contribute to the pathogenesis of the complex phenotypes described here. Moreover, as the missense variants that we tested did not display any obvious stability issue upon overexpression, dominant negative effects cannot be excluded. Finally, second hits or blending phenotypes with additional variants in other (known or unknown) genes associated with the clinical phenotypes described in this study could contribute to the observed clinical variability.

Patients 11-I and 15-I carried the same protein-elongating p.Pro1343Glnfs*49 variant. Although this variant occurs too distally in the messenger RNA (mRNA) sequence to induce nonsense-mediated decay, its pathogenicity is supported by the fact that this variant, together with the previously described p.Glu1344Lysfs*49 variant,^18^ all occurred de novo in similarly affected individuals. Although some of the patients in this series carried additional rare variants, none of these are likely pathogenic. Individual 1-I carries a 441-kb heterozygous interstitial microduplication in 22q11.2 (hg19 (chr22:22,817,344–23,258,369) that does not contain any disease-associated genes and was inherited from his healthy father. Individual 5-I carried the recurrent 312-kb interstitial duplication at 15q11.2 (hg19: 22,770,421–23,082,328) that was inherited from her healthy mother. In a large study on the effect of copy-number variants on cognition, 136 control individuals carrying the 15q11.2 duplication performed to a similar level as population controls on all tests of cognitive function.^34^ In family 7, where both mildly affected siblings (individuals 7-I and 7-II) inherited the *NSD2* variant from their similarly affected mother, we additionally found that individual 7-I carries a paternally inherited 783-kb deletion at 9p13.3 (hg19: 35,487,232-36,270,255) as well as the maternally inherited *GABBR2* c.1656_1657delTT, p.Cys553Hisfs*96 variant. Her father had learning disability and depression. No overlapping deletion was present in the DECIPHER database but, based on the gene content and on the family history, a modulatory role of the 9p13.3 deletion on her phenotype cannot be excluded. Except for one truncating variant described in a large sequencing study on patients with neurodevelopmental delay,^35^ all *GABBR2* pathogenic variants described until now in this gene are missense (source: HGMD Professional, release 2020.2, see Web Resources). This, together with the fact that her similarly affected brother (individual 7-II) did not carry the p.Cys553Hisfs*96 *GABBR2* variant, speaks against a determining role on the phenotype. Individual 10-I carries a 38-kb partial *HUWE1* duplication in Xp11.2, which overlaps with a recurrent polymorphic copy-number variant that does not affect the expression of *HUWE1* and thus should have no effect on cognition.^36^

Our study leaves unanswered questions for future research, such as the clarification of the full molecular mechanisms. This may reveal potential targets for therapeutic interventions relevant not only to patients with *NSD2* deficiency, but also potentially even to patients with obesity in general.

In conclusion, our data support the concept that *NSD2* loss of function is associated with a distinct phenotype that only partially overlaps with WHS and constitutes a differential diagnosis to Silver–Russell and similar syndromes. This phenotype is in line with the distinct features described in the first patient with a small deletion encompassing *NSD2* by Rauch et al.^8^ This patient now at age 26 years is 172 cm tall (-0.55 SDS), weighs 45–50 kg (ca. -2.5 SDS), and has a “small head.” Despite his normal eating behavior, all attempts to gain weight have been unsuccessful. He has an IQ in the lower-normal range, a calm and content personality, and lives with his parents. As a reflection of the distinct facial gestalt and the greatly different disease severity, especially concerning ID, of *NSD2* deficiency and contiguous gene deletions leading to WHS, which may also be important for families’ and physicians' perception of the patients’ prognosis, the *NSD2*-related disorder may be named Rauch–Steindl syndrome after the delineators of this phenotype.^8^

## Web Resources

DECIPHER: https://decipher.sanger.ac.uk

DSM-5 severity levels for intellectual disability: https://dsm.psychiatryonline.org/doi/full/10.1176/appi.books.9780890425596.dsm01

Face2Gene: https://www.face2gene.com/

GeneMatcher: https://genematcher.org

gnomAD: https://gnomad.broadinstitute.org/

Growth curves of the Swiss society of pediatrics: https://www.kispi.uzh.ch/de/zuweiser/broschueren/Seiten/document.axd?id=56c0bb56-1793-48f3-968e-238915f47bbd9

HGMD Professional: http://www.hgmd.cf.ac.uk/ac/index.php

HPO: https://hpo.jax.org/app/

OMIM: http://www.omim.org/

PDB: https://www.wwpdb.org

RasMol: http://www.openrasmol.org/

Smart: http://smart.embl-heidelberg.de/

SwissModel: https://swissmodel.expasy.org/

Uniprot databank entry for NSD2: https://www.uniprot.org/; Entry O96028

## Supplementary information

Supplementary Data

Supplementary tables

## Data Availability

All materials, data sets, and protocols presented in this work are available upon request to the corresponding authors. *NSD2* variants have been deposited in the DECIPHER database (see Web Resources) under the following IDs: 422873, 422875, 422876, 422877, 422878, 422879, 422880, 422881, 422882, 422883, 422885, 422886, 422887, 422888, 422889, 422890, 422891, 422892.
