# Supplementary material for: Loss-of-function and missense variants in NSD2 cause decreased methylation activity and are associated with a distinct developmental phenotype
Source: Genet Med. 2021 May 3;23(8):1474–83. doi: 10.1038/s41436-021-01158-1 (PMC8354849; doi:10.1038/s41436-021-01158-1)
Supplement: Supplementary file 1 — Supplementary Data [file 41436_2021_1158_MOESM1_ESM.pdf]

Supplemental Data

Figure S1

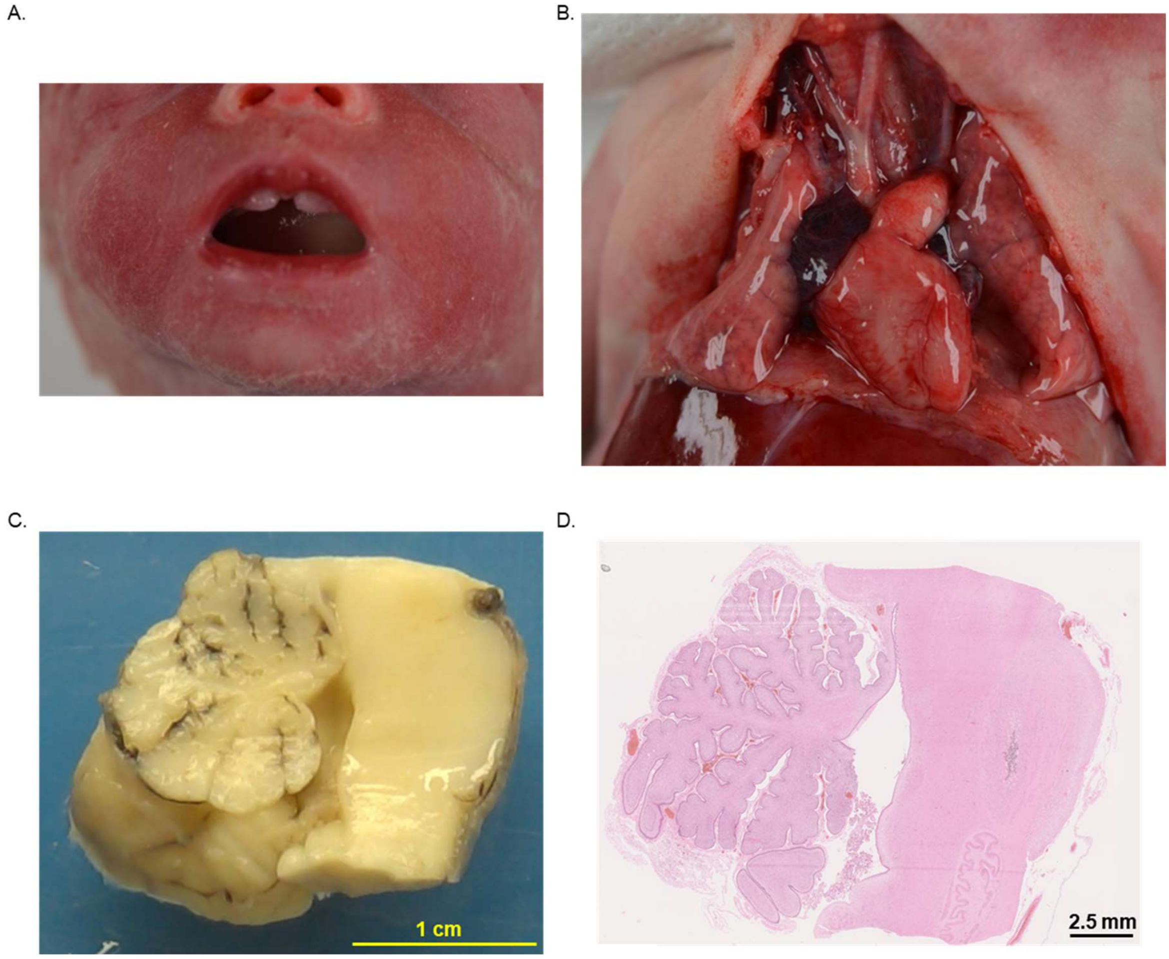

Postmortem pathological findings in patient 16-I. A. Median superior gingival cleft. **B.** Interrupted aortic arch type B. The interruction occurs near the emergence of the left carotid artery. No residual fibrous strands were visible. **C** and **D.** Gross (**C**) and microscopical (**D**) pathology of the patient's cerebellum. Notice the hypoplasia of the vermis.

Supplemental Note: Case Reports

Patient 1-I is an 18-year-old Caucasian boy who was referred at the age of 26 months for failure to thrive, mild motor delay and muscular hypotonia. The affected individual was born at 41 weeks of gestation via vaginal delivery from a 37-year-old mother and a 41-year-old non consanguineous father. During pregnancy a diagnosis of intrauterine growth retardation was made. He presented with low body weight (2480g, -2.79 SDS) and short length (45 cm, -3.52 SDS). In the neonatal period he had feeding difficulties and received a diagnosis of failure to thrive at the age of 12 months. All motor milestones were mildly delayed, with unaided walking being achieved at the age of 18-19 months. Speech development was also mildly delayed. His IQ was 78 to 81 at the age of 11 years. At the age of 18 years the patient could express himself fluently in his native language. He successfully completed an anthroposophical "Steiner" school with the support of a learning coach and subsequently started a vocational school for horticulture. He is autonomous for all daily activities, including driving a moped as well as a tractor. A bone age determination according to Greulich and Pyle showed a bone age of 9.25 years at the chronological age of 10 years and 6 months. Dental crowding was successfully treated with orthodontic braces. At the age of 11 years he received psychological support after expressing suicidal thoughts related to difficulties at school. At the age of 18 years these behavioural abnormalities had receded and he was happy and social. At the last visit he presented with underweight (45.4 kg, -2.78 SDS). His height (164.5 cm, -1.56 SDS) and head circumference (55 cm, -1.34 SDS) were in the lower-normal range. The patient showed mild facial anomalies including triangular face, broad forehead, protruding ears and high arched palate. Furthermore, he presented with pectus excavatum, scapulae alatae, mild scoliosis, prominent knees, broad forefeet, bilateral clinodactyly of the 5th toe and flat feet. A methylation-sensitive MLPA for Silver-Russel syndrome as well as the direct sequencing of PTPN11 and SMARCA2 were negative. A chromosomal SNP-microarray analysis showed a paternally inherited 441 kb interstitial duplication at 22q11.22 (22817344-23258369). Trio-exome sequencing revealed the c.2606G>A, p.Cys869Tyr *de novo* variant in *NSD2*.

Patient 2-I was the 2-year-old son of a 34-year-old 7th gravida 2nd para Caucasian mother and a 36-year-old non consanguineous Caucasian father, who was referred to us at the age of 12 months with short stature, agenesis of the left kidney, a mild peripheral stenosis of the left pulmonary artery, recurrent infections and digestive problems. During pregnancy he was small for gestational age, growing proportionally at the 3rd percentile. A NIPT test performed because of increased nuchal translucency showed no signs of chromosomal imbalances. In addition, fetal ultrasound at 18 weeks of gestation raised the suspicion of an agenesis of the left kidney in combination with esophageal atresia. The former but not the latter was subsequently confirmed. He was born at

39 weeks of gestation via planned C-section. At birth he presented with low body weight (2390 g; -2.53 SDS), short length (48 cm; -1.7 SDS) and microcephaly (32 cm; -2.54 SDS). His APGAR scores were 7/8/10 and his primary adaptation was uncomplicated. At the age of 6 days he developed jaundice (bilirubin 252umol/l) that was treated with phototherapy. Shortly after he was hospitalized because of failure to thrive with a weight loss of 190g since birth and a milk intake of 180ml/kg/day. While hospitalized the agenesis of the left kidney was confirmed sonographically and the pulmonary artery stenosis was first diagnosed. An urinary organic acid determination in the neonatal period showed a mild increase in the elimination of methylmalonic acid. At the age of 7 months his plasma methylmalonic acid levels were determined to be 2620 nmol/l (normal range 73-271nmol/l). The first year of life was complicated by the persistence of feeding difficulties as well as recurrent infections, including a severe RSV infection at the age of 3 months that required hospitalization in the ICU. He started shuffling at the age of 8 months and walking unaided at 16 months. He spoke his first words at 24 months of age. At the age of 2 years and 2 months he presented with short stature (75.7 cm, -4.13 SDS), underweight (8.84 kg; -3.05 SDS) and microcephaly (46.5 cm; -2.71 SDS). He spoke 5-6 words and had a tendency to constipation. He had a broad and prominent forehead, a bulbous tip of the nose, retrognathia and low set posteriorly rotated ears. Bone age assessment showed a 6 months delay. His renal function parameters were always normal. MLPA for the SHOX gene as well as a screening for the most common CFTR mutations were negative. Whole-exome analysis revealed the c.1569dup, p.Lys524Glufs*17 *de novo* variant in *NSD2*. An additional filter for metabolic disorders did not reveal variants which could explain the elevated methylmalonic acid levels in plasma.

Patient 3-I is a 50-year-old Caucasian female patient who was referred first twenty years ago for evaluation of severe global developmental delay, facial dysmorphism, stereotyped and aggressive behavior and postnatal growth deficiency. She was born at 40 weeks of gestation via vaginal delivery without fetal distress from a 23-year-old mother and a 27-year-old father. The pregnancy was clinically uneventful. At birth she presented with low body weight (2170 g, -3.09 SDS), short length (48 cm, -1.68 SDS), and hypotonia. Unaided sitting was achieved at 8 months, unaided standing at 14 months and unaided walking at 18 months. She never acquired speech except for a few words and at the time of the last visit was not autonomous in feeding and dressing and was not toilet trained. The behavioral abnormalities receded after the age of twenty. At the age of 50 she presented with short stature (height 148 cm, -2.28 SDS), microcephaly (OFC 48.5 cm, -6.34 SDS) and overweight (body weight 60 kg, +0.24 SDS; BMI 27.4 kg/m^2, +1.59 SDS). She presented also with a complex progressive eye phenotype with keratoconus, retinitis pigmentosa and optic nerve atrophy. The family history

revealed an isolated keratoconus in her otherwise not affected sister. The patient presents facial dysmorphism including high forehead, deeply set eyes, bulbous tip of the nose, short philtrum and prominent upper central incisors. Her hands and feet are short with tapering fingers. At first Cohen syndrome was suspected, but *VPS13B* direct sequencing as well as a search for rearrangements revealed no abnormalities. Neutrophil counts as well as a brain MRI were also normal. Karyotyping and array-CGH analysis showed a normal female karyotype. Exome sequencing analysis revealed the NM_001042424.2, c.3223_3226dup, p.(Gly1076Valfs*16) frameshift variant in the *NSD2* gene. The variant was not detectable in her unaffected mother and sister. Her unaffected father could not be tested. Furthermore, we applied a filter for retinitis pigmentosa, keratoconus and optic nerve atrophy to the exome sequencing data to determine whether the patient carried additional variants that could explain her ocular phenotype. This additional analysis showed 3 heterozygote variants of unknown significance in RHO (NM_000539.3:c.192G>T, p.Gln64His), KRT3 (NM_057088.2:c.14C>T, p.Ala5Val) and UNC45B (NM_001033576.1:c.955C>T, p.Arg319Cys). A segregation analysis determined that all three variants were maternally inherited and are carried by the sister with keratoconus as well, suggesting that at least one of them may be associated with an ocular phenotype with incomplete penetrance.

Patient 4-I is a 6-year-old Caucasian male. He was adopted at age 4 years from an orphanage in Bulgaria and was severely malnourished at the time of adoption. Information on his neonatal history is sparse, but he is thought to have been born prematurely, to have suffered a neonatal pneumothorax and to have spent about one month in the NICU. His developmental milestones have been severely delayed and independent ambulation was not achieved until the age of 5 years. He suffered a dural venous sinus thrombosis at age 4 years that was successfully treated with enoxaparin. An MRI of the brain showed a periventricular leukomalacia, consistent with his history of premature birth. At the age of 6 years he had not yet developed verbal communication, was not toilet trained and was dependent for all cares. He received special education and his demeanour was described as affectionate and anxious, with autistic features such as sensory defensiveness and hand flapping. Cerebral MRI showed T2 hyperintensities and volume loss of the periatrial white matter. He was fed exclusively via a gastrostomy at a slow rate because of a feeding intolerance with retching and vomiting. His weight was 12 kg (-4.37 SDS), height was 99 cm (-3.44 SDS), OFC was 44 cm (-6.3 SDS) and BMI was 12.2 kg/m^2 (-2.88 SDS). He had dolichocephaly, high forehead, bushy eyebrows, exotropia, a prominent tip of the nose, low-set ears and widely spaced prominent eyes. Chromosomal microarray analysis showed no imbalance. Singleton-exome analysis revealed the *NSD2*, c.1588_1589dupAA, p.Ile532Glyfs*67 heterozygote variant. No segregation analysis was performed as DNA samples of the biological parents were not available.

Patient 5-I was a 5-year-old female born by vaginal delivery at 37 weeks of gestation to a 29-year-old 6th gravida 3rd para Jordanian mother and a non-consanguineous father with Jordanian and Syrian origins. The delivery was induced secondary to CTG-decelerations. Her Birth weight was 1980 g and her Apgars were normal. The pregnancy was complicated by intrauterine growth retardation and a pregnancy-associated thrombotic disorder treated with aspirin and heparin. An amniocentesis was normal. The patient presented with feeding difficulties at birth and by four months she was refusing food by mouth. A nasogastric tube was placed after a video fluoroscopic swallowing study revealed silent aspiration. At age 13 months she was diagnosed with failure to thrive and received a gastrostomy tube. By age 3, she started to feed orally and became less dependent on the G-tube. Her psychomotor development was delayed from birth, with steady progress and no history of regression. Social smiling was achieved at the age of 6 months, unassisted sitting at 12 months, unaided standing at 15 months and unaided walking at 18 months. She started speaking at the age of 30 months. A previously diagnosed hemiplegic infantile cerebral palsy was not confirmed at the age of 4 years, when she showed symmetric muscle tone. At the age of 4.5 years she was not yet toilet trained. A first brain MRI at the age of 28 months revealed an 8 mm pineal cyst that was considered as an incidental finding. A second brain MRI at the age of 50 months showed non-specific mild diffuse T2/FLAIR signal hyperintensities in the bilateral parietal and occipital white matter. A spinal MRI showed a small thoracic spinal cord syrinx (ddx prominence of the central canal). As a newborn she failed an otoacustic emission test as well as a follow-up test. An auditory brainstem response test at the age of 9 months showed nevertheless no significant abnormality. A cardiological examination showed a small, hemodynamically not significant PFO and an electrocardiographical left axis deviation with normal sinus rhythm. Possible signs of a precocious puberty were noted at around 15 months and only consisted of a few dark hairs present at labial cleft. Otherwise, she had a normal female exam. The precocious puberty has been non-progressive. She did have a full endocrine workup which was normal and was discharged from further endocrinology follow up. Physical examination at age 5 years revealed a petite female with stature in the lower-normal range 105.2 cm (-0.89 SDS), underweight (13.01 kg, -2.41 SDS; BMI: 11.8 kg/m^2, -2.88 SDS) and microcephaly (48 cm, -2.2 SDS) who was friendly and cooperative. Her demeanour was described as social with a tendency towards overreaction, screaming and crying fits. She attends a specialized pre-school and shows poor retention and difficulty following 2-step directions. The parents reported occasional gasping for breath during sleep. Her neurological examination was normal. The patient's father has no known medical problems. The patient's mother has a history of three prior pregnancy losses, including a six-month stillborn male. The family history was otherwise negative, including two older unaffected half-sisters.

Chromosomal microarray analysis revealed the recurrent 312 kb microduplication at 15q11.2 (22,770,421-23,082,328) inherited from the healthy mother and a single long contiguous region of homozygosity at 1p13.1q21.3(117,507,792-154,668,893). Trio-exome sequencing showed the c.3271G>A, p.Glu1091Lys *de novo* variant in *NSD2*.

Patient 6-I is a 10-year-old Caucasian patient referred for the first time at 3 years for muscular hypotonia and global developmental delay. He is the son of a healthy mother and an affected nonconsanguineous father (patient 6-II). Patient 6-I was born at 41 2/7 weeks of gestation. His birth parameters were as follows: length 49 cm (-1.83 SDS), weight 3410g (-0.84 SDS), OFC: 38.2 cm (+1.5 SDS). He had mild neonatal hypotonia and feeding difficulties but did not require intensive care. He had bilateral cryptorchidism, and the right atrophic testis was surgically removed at the age of 12 months. All motor milestones were delayed, with unaided walking being achieved at the age of 37 months and with the first words being pronounced at the age of 36 months. At first clinical exam at the age of 2 years 11 months, his height was 89 cm (-1.06 SDS), his weight 11.050 kg (-1.65 SDS), and OFC was 50 cm (-0.28 SDS). He had a triangular face, with a large and high forehead, and with a small pointed chin, short and upslanted palpebral fissures, mild hypotelorism and arched eyebrows. His voice was nasal. He had small joints hyperlaxity, clinodactyly of the 5th fingers, flat feet and overlapping 1st and 2nd toes. At neurological exam, he had axial and peripheral hypotonia and wide-based gait. At that time, chromosomal rearrangements, Fragile X syndrome and Steinert disease were excluded. CK dosage and thyroid function were normal. At the age of 51 months, bone age was delayed (estimated at 32 months). At the age of 5 years, he still had motor delay. He could not jump with both feet and still had difficulties climbing stairs. He also had fine motor difficulties. He used a large vocabulary but had difficulty with pronunciation. A hearing test showed no abnormalities. He had no behavioral issues. He still presented with height below the norm and underweight (height 96 cm, -2.72 SDS; weight 13.800 kg, -1.96 SDS) but normal OFC (51 cm, -0.42 SDS). Abnormal 11p15 methylation, uniparental disomy of chromosome 7 and a mutation in the hotspot region of the SRCAP gene were excluded at this timepoint. At the age of 6, he still complained about generalized muscular weakness, with difficulties for his day-to-day life. He was oriented to a special school. An IQ evaluation showed disharmonic scores (WIPPSI III: verbal IQ 89, performance IQ 63). At 10 years, disharmonic performances were confirmed (WISC-V: verbal comprehension index 92; visual spacial index 64; fluid reasoning index 69; working memory index 76, proceeding speed index 83). He could read simple sentences, but graphism was still difficult due to severe dyspraxia. Simple arithmetic operations remained very difficult. Clinical examination was very similar to that previously described with global hypotonia, triangular face, slight

facial dysmorphic features, small joints hyperlaxity, flat feet and bilateral 5th fingers clinodactyly. Patellar deep tendon reflexes were slightly increased. Heart ultrasonography was normal. Body measures at the age of 10 years were as follows: height 133.5 cm (-1.05 SDS), weight 26.3 kg (-1.33 SDS), OFC: 53 cm (-0.61 SDS), BMI: 14.8 kg/m^2 (-1.29 SDS). His father (patient 6-II) has triangular face with early temporal baldness, arched eyebrows, hypotelorism, nasal voice and retrognathia. He attended a special school and presents with mild intellectual disability (total IQ 52). A trio-clinical exome sequencing allowed the identification of the c.3472dup, p.Asp1158Glyfs*11 paternally inherited variant in *NSD2*. This variant was also found in another affected family member. This boy, his obligate carrier mother, and two other members of the family shared similar phenotypic characteristics as patients 6-I and 6-II but did not consent to collaborate to this study (Figure 4C).

Patient 7-I is now 13 years old Caucasian female who exhibits developmental delay, ADHD, other behavioral abnormalities, and microcephaly. The patient was born full term via spontaneous vaginal delivery without perinatal complications. There were no maternal illnesses or exposures during pregnancy. She was initially referred to gastroenterology at 2 ½ years of age for evaluation of poor growth and was noted then to have delayed developmental milestones. Her workup was unremarkable except for a paternally inherited 9p13.3 microdeletion of unclear clinical significance (see Table S3 and Discussion). The patient was later referred to genetics for further evaluation. She started walking at 18 months but continued to be clumsy until 3-4 years of age. She started to say single words around 18 months of age and short phrases at 3 years old. She received therapeutic services for her delayed development. She later continued to exhibit poor communication skills and started exhibiting behavioral difficulties including some aggressive behaviors and stubbornness. She was diagnosed with attention deficit hyperactivity disorder (ADHD) treated with lisdexamfetamine, with some improvement. She attends special education classes and has an Individualized Education Program in place. Her physical examination showed microcephaly, small epicanthal folds bilaterally, posteriorly rotated ears that were simple with hypoplasia of lower two third of helices, short nose, thin upper lip and multiple teeth capped due to severe decay. In addition she had inverted nipples and 2-3 toes cutaneous incomplete syndactyly bilaterally (found in her father). Her diagnostic workup included a chromosomal microarray, which revealed a paternally inherited ~800 Kb deletion on 9p13.3 (chr9:35487232-36270255; hg19). Acylcarnitine profile, total/free carnitine, serum amino acids, lactate, ammonia, urine organic acids, urine amino acids, CK, CDGs, CBC, CMP, celiac panel, cholesterol, urine oligosaccharides, thyroid function, all were normal. Her exome sequencing

revealed two maternally inherited heterozygous variants of uncertain significance: in *NSD2* designated as c.2684C>T, p.Pro895Leu and in *GABBR2* designated as c.1656_1657delTT, p.Cys553Hisfs*96.

Patient 7-II, the brother of 7-I, is 10 years old and exhibits poor weight gain, microcephaly, developmental delay and ADHD. He was born full term via C-section with no perinatal or neonatal complications. Mother had no exposures or illnesses during pregnancy. Currently he continues exhibiting pronunciation problems, for which he receives speech therapy, and some of fine motor skill difficulties including buttoning and zipping. He is enrolled in special education classes for most subjects and has an Individualized Education Program in place. He cannot read and still learning to recognize letters and numbers. He can do basic self-care skills if prompted. He was diagnosed was ADHD. His physical examination showed microcephaly, thin body habitus, triangular face, broad forehead, broad nasal bridge, mild posterior rotation of ears, and small ear lobes. The remainder of physical examination was unremarkable except for -3 toes cutaneous incomplete syndactyly bilaterally. His diagnostic workup included normal chromosomal microarray, ammonia, serum amino acids, acylcarnitine profile, urine organic acids, urine amino acids, and CDGs. Thyroid function studies, Celiac panel, CBC, CMP, CK, immunoglobulins, IGF1, and IGFBP3 all were normal. Skeletal survey demonstrated 11 ossified ribs and 6 non rib-bearing lumbar vertebrae but no osseous abnormalities. Bone age at 30 months old and 4 years old was delayed. His exome sequencing revealed a heterozygous maternally inherited variant of uncertain significance in the NSD2 gene: c.2684C>T, p.Pro895Leu.

The mother of the two siblings needed special education classes for English and math but completed high school. She has significant teeth decay (enamel hypoplasia?). She had a thin body habitus as a child. Her height was 160 cm (-0.46 SDS) and her OFC was 53.5 cm (-1.46 SDS). The father of the two siblings had learning disabilities, depression, and hypertension. Both parents are of Caucasian descent. The siblings have an 11-year-old brother with normal development and growth.

Patient 8-I is the daughter of two nonconsanguineous Caucasian parents. She was born by C-section at 38 2/7 weeks of gestation with a birth weight of 2630 g (-1.7 SDS). IUGR was diagnosed at 20 weeks of gestation. At birth she presented with feeding difficulties and gastroesophageal reflux. At 17 months she could walk while holding on to table. Developmental testing using the Alberta Infant Motor Scale (AIMS) and the Bayley Scales of Infant Development (BSID) scales showed psychomotor development below the 5th percentile. The patient is a content and quite child. Eye contact was initially poor but later improved. At the age of 16 months her growth parameters were as follows: height 73 cm (-2.01 SDS), weight 7.9 kg (-1.83 SDS), OFC 43.8 cm (-2.59 SDS),

BMI 14.8 kg/m2 (-1.04 SDS). A brain MRI at the age of 10 months showed no structural abnormalities. A heart ultrasound as well as an ECG were anamnestically normal. Basic metabolic testing was negative. She presents with mild hypotonia, a mild synophrys, upwards slanting palpebral fissures and 1 café au lait spot. Array-CGH was normal. Whole exome sequencing allowed the detection of the c.1103_1104del, p. Glu368Valfs*13 variant in *NSD2*.

Patient 9-1 is 9 years and 10 months old girl who was born after a normal pregnancy of 42 weeks with a birthweight of 2770 gram (-1.93 SD) to two nonconsanguineous Caucasian parents. She presented with feeding difficulties in the first two weeks of life. Weaning started at the age of 8 months and was also problematic. At 2 months, ultrasound of the abdomen was performed because of a short perineum and no abnormalities were noted. Her weight at 9 months was -2.5 SD and her development was noted to be delayed: she started crawling at 16 months and walking unaided at 2.5 years of age. At the age of 2 years a delay of 9 months was assessed. Physical examination at the age of 2.5 years showed a height of 80 cm (- 3.03 SDS), weight 10.3 kg (-1.72 SDS), and a head circumference of 47 cm (-1.7 SDS). She had thin hair, coarse facial features, strabismus divergence of the left eye, upward slant palpebral fissures, mild hypertelorism, iris stellatae, low broad nasal bridge, elevated philtrum, widely spaced small teeth, broad mouth with full lips. She presented also with tapering fingers, 5th finger clinodactyly and joint hyperlaxity (Beighton score 6/8). Metabolic and chromosome analysis (250K SNP array) did not reveal any abnormalities. The girl was seen again in the genetic clinic at the age of 9 years and 10 monts. Her total IQ was estimated at 69. She had a height of 123.5 cm (-2.22), a weight of 21,4 kg (-2.07 SDS) and a head circumference of 51 cm (-1.3 SDS). Her BMI was 14 kg/m2 (-1.52 SDS). Similar facial features were noted as described above. A Trio-WES revealed the c.2160T>A, p.Cys720* *de novo* variant in *NSD2.*

Patient 10-I was first seen at the age of 4 years and for the last time evaluated at the age of 12 years. He was born as the second child of healthy non-consanguineous parents at a gestational age of 41 5/7 weeks with a birth weight of 2720 g (-2.32 SDS). The pregnancy was uneventful, however mother reported that she felt very few fetal movements and there was few amniotic fluid at birth. The boy was born with club feet and flexion contractures of arms and legs that resolved after conventional treatment. The boy experienced feeding problems during the neonatal period. He developed epilepsy at the age of 1 year, triggered by fever. The EEG showed epileptic phenomena that successfully responded to anti-epileptics. After a convulsion-free period, anti-epileptics were stopped at the age of 7 years. His growth, hearing and vision were normal, but he showed a delay in motor and speech development. At the age of 3 years his IQ was estimated at 54. At the age of 6 years,

parents told that he was sensitive to changes, had his own rituals and preferred to play alone. At the age of 7 years he was formally diagnosed as having an autism spectrum disorder. His IQ remained around 50 during subsequent tests. The boy's elder sister is healthy. Father reported to have had convulsions at a young age. At physical examination no dysmorphic features were seen, apart from a thin upper lip and mild shawl scrotum. Whole exome sequencing revealed a *de novo* c.3410C>T p.(Ser1137Phe) in *NSD2*.

Patient 11-I was first seen at the age of 18 months. He is the second child of healthy non-consanguineous parents. He was born at a gestational age of 38 weeks by Cesarean section because of intrauterine growth restriction due to a bi-lobar placenta. His birth weight was 2195 g (-2.56 SDS). Birth length was 42 cm (-3.96 SDS). He suffered from failure to thrive with feeding problems and recurrent airway infections. He had low levels of IgA and IgG3. He showed a delay in motor and speech development, as well as a delayed visual maturation. Hearing, MRI, EEG and SNP-array were normal. His older brother is healthy and the family history is negative for developmental delay. At physical examination a mild epicanthus and prominent forehead were seen. The boy makes good contact, is friendly and happy. Exome sequencing revealed a *de novo* varaiant c.4028delC, p.(Pro1343Glnfs*49) in *NSD2*, that is predicted to resulted in a frameshift in the last exon (exon 24) with elongation of the protein (26 extra amino acids).

Patient 12-I is the son of two nonconsanguineous Caucasian parents. He was born after an uncomplicated pregnancy of 40 weeks with a birth weight of 4250 g (1.41 SDS). Birth length was 53 cm (0.22 SDS) and OFC was 38 cm (1.85 SDS). In the neonatal period, an aberrant shape of the head was noted, and patient was diagnosed with craniosynostosis, for which surgery was performed at the age of 6 months. The patient had motor delays and was reported to be clumsy. He is currently at an adult age and has a mild intellectual disability. In addition, he has been diagnosed with autism spectrum disorder and has anxiety problems. The patient is currently living on his own with some support and works as a graphical designer in an environment adjusted for people with autism spectrum disorders. He suffers of severe joint problems and was recently diagnosed (around age 30y) with severe arthrosis in his knee and wrist. A Trio-WES analysis revealed the c.3056A>G; p.Lys1019Arg heterozygous *de novo* variant in *NSD2* as well as the *AGO2* NM_012154.3: c.1810G>A; p.Gly604Arg heterozygous *de novo* variant.

Patient 13-I is a 6-year-old male who was first referred to a genetic clinic at 9 months of age because of poor weight gain and developmental delay. He is the son of a 38 years old mother of English and Trinidadian background and a 46 years old Caucasian father. The parents were nonconsanguineous. He was born at 41

weeks of gestation by C-section due to failure to progress. He presented with short length (48.2 cm, -2.13 SDS). Birth weight and head circumference were in the normal range (3350 g, -0.86 SDS and 35 cm, -0.69 SDS, respectively). No concerns were noted during the pregnancy. An amniocentesis performed because of advanced maternal age showed a normal male karyotype. Early concerns were related to poor weight gain and developmental delay, most notably in the area of motor development. These issues have continued to be a consistent finding up to the last visit. He could sit unassisted at 9 months, could walk unaided at 15 months and said his first words also at 15 months. Speech delay has been for him a persistent issue. At 6 years of age he attends regular classes in a kindergarten although with difficulties. His motor skills are appropriate for age. His IQ is estimated to be in the low normal range (IQ~89 at 6 1/2 years). He is a happy, appropriately social child and presents hand flapping when excited. His sleep is disturbed, and he wakes up most nights despite receiving melatonin. A brain MRI at the age of 2 ½ years was normal except for few small areas of gliosis in the frontal and periatrial area. He was seen by endocrinology due to mild short stature. Bone age was mildly delayed. He has a history of hypoplastic tooth enamel and has had several dental implants due to cavities. He presents also mild myopia. At 6 years of age, his height was 108 cm (-1.61 SDS), weight was 14.4 kg (-2.8 SDS), and head circumference was 50 cm (-1.66 SDS). He had a triangular face shape, broad forehead, wide set eyes with horizontal palpebral fissures, slightly protruding ears, slightly short philtrum and mild micrognathia. Muscle tone, which was found to be reduced until the age of 4, was appropriate for age. A chromosomal microarray found a 69 kilobase deletion at 2q21.2 (containing only one gene, namely *NCKAP5*) and an 872 kilobase duplication at Yp11.2. A Duo-Exome sequencing identified c.3547delT, p. Cys1183Valfs*146 in *NSD2* which was not inherited from the mother.

Patient 14-I is the second child of non-consanguineous French parents. She has a healthy 12-year-old sister who had feeding difficulties until the age of 3. IUGR was first diagnosed in the proband in the 3rd trimester. She was born at 40 weeks of gestation by uncomplicated vaginal delivery. Measures at birth were as follows: length: 45 cm (-3.05 SDS), weight: 2600g (-2.07 SDS), OFC: 32 cm (-2.23 SDS); Apgar was 10-10. The proband presented feeding difficulties since birth, which led to a genetic evaluation at the age 7 months because of failure to thrive. Enteral feeding by nasogastric tube was initiated at the age of 6 months and a gastrostomy was performed at the age of 4 years 8 months. She could sit at 9 months and walk independently at the age of 19 months. Speech development was delayed. She said her first words at 3 years old. At 6 years old, she did not use "I" and couldn't build sentences correctly. Speech and language therapy were started at an early stage. The mother reported interaction issues with her daughter, and social smiling appeared only at the age of 1 year and 6

months. Urinary continence was acquired at 3 years, but an encopresis persisted until the age of 5 years. Cerebral MRI and EGG, both performed at 4 years 5 months, were normal. An abdominal ultrasound as well as a hematochemical panel (including a metabolic screening, transaminases, ionogram, complete blood count, PT, PTT, platelet count, fibrinogen, calcium, phosphorus, vitamins B9 and B12, IgAs and anti-transglutaminase antibodies) did not show any abnormalities. The patient received supplements for zinc and iron deficiency in infancy. Growth parameters at last examination (age: 8 years 8 months) were as follows: height: 121.5 cm (-1.5 SDS) / weight: 18.8 kg (-2.12 SDS), OFC: 49.3 cm (-2.44 SDS), BMI: 12.7 kg/m^2 (-2.29 SDS). She has a relative short and smooth philtrum, micrognathia, dental diastasis, enamel hypoplasia, protruding ears, enhanced protrusion of the inferior crus (relative to the prominence of the antihelix stem), everted antitragus, small ear lobes and generalized hypotonia. She attents the normal school system and receives speech therapy. A developmental evaluation did not show any intellectual disability (Verbal comprehension index: 100; Visual spatial index: 86; Fluid reasoning index: 85; Working memory index: 82; Processing speed index: 116). Although her intellectual abilities are in the average range for her age, she has difficulties with logical reasoning. On the other hand, she has very good memory skills in both auditive and visual modalities. A speech and language assessment showed an articulation disorder, phonological difficulties and a language disorder (receptive and expressive) associated with a sensory dysorality syndrome. A Trio-"mendeliome" (panel of 3989 genes involved in neurodevelopmental impairment) identified a heterozygous truncated variant in the exon 11 of *NSD2* (NM_133330.2): chr4:g.1941422C>T ; c.1798C>T ; p.(Arg600*).

Patient 15-I is a 4 year old boy, the first child in the family. He was born at 38 weeks of gestation by Cesarean section. Measures at birth were as follows: length: 38 cm (-5.7 SDS) / weight: 2312 g (-2.29 SDS) / OFC: 32 cm (-2.07 SDS); Apgar score 8/9. At the 34. week of gestation intrauterine growth retardation and left hydronephrosis was diagnosed by ultrasound. His early development was delayed; he started turning at 6 months and sitting at 12 months. He has mild facial dysmorphism (triangular face, prominent forehead, palpebral fissures slanted up, micrognathia), muscular hypotonia, depigmentation of the skin along the Blascho lines on both hands and 5th finger clinodactyly. At the age of 5.5 months vitamin B12 deficiency was diagnosed and treated (151 pmol/l, normal 216 - 893). Statural growht is also delayed. At the age of 16 months his height was 75 cm (-2.02 SDS), weight 8.2 kg (-2.25 SDS), and OFC 45 cm (-2.59 SDS). A brain MRI at the age of 2 years and 3 months showed a thin corpus callosum as well as white matter lesions. At 3 years of 4 months of age his cognitive abilities corresponded to 20 months and expressive speech to 12 months. His developmental delay was approximately 1.5 years. On the last examination at the age of 4y 4m, his height was 104 cm (-0.36 SDS),

weight 15 kg (-0.98 SDS), and OFC 50 cm (-1.05 SDS). He could say 3-4 words and more syllables and could eat independently. An EEG showed epileptic activity in the absence of clinical seizures. He is hyperactive and avoiding eye contact. Bifid uvula was additionally detected, which associates to irritability when eating solid foods. Trio-WES identified the c.4028delC, p.Pro1343Glnfs*49 *de novo* variant in *NSD2*.

Patient 16-I was a male fetus whose pregnancy was terminated at the 26 6/7 week of gestations because of ultrasonographic anomalies. An ultrasound in the 22nd week of gestation revealed intrauterine growth retardation, microcephaly and a complex heart malformation with interrupted aortic arch and a ventricular septal defect. A prenatal array-CHG analysis showed no chromosomal imbalances. The measures at termination were: length: 32 cm (-1.11 SDS), weight: 710 g (-1.3 SDS), OFC: 23 cm (-1.5 SDS). An autopsy revealed a cerebellar malformation with vermis hypoplasia and rudimentary anterior leaflets, an interrupted aortic arch type B as well as a median superior gingival cleft. Hypertelorism was noticed as well. A Trio-WES allowed the detection of the c.2263C>T, p.Arg755* *de novo* variant in *NSD2*.

Supplemental Tables

Table S1 (provided as Excel file). *NSD2* variants published in previous large-scale sequencing efforts.

Footnotes to Table S1: [a]All cDNA and protein positions refer to the NM_133330.2 NSD2 transcript

Table S2 (provided as Excel file). List of the reagents used in the *in vitro* assays.

Table S3 (provided as Excel file). Detailed clinical data for the 18 patients carrying *NSD2* variants characterized in this study.

Footnotes to Table S3:

All cDNA and protein positions refer to the NM_133330.2 *NSD2* transcript

L: length; W: weight; H: height; BMI: body mass index; OFC: occipitofrontal circumference; GW: gestational week; ID: intellectual disability; NA: not available; OT: occupational therapy; ST: speech therapy

[a]The mother did not carry the p.Gly1076Valfs*16 variant, the father could not be tested. Both parents were not affected.

[b]Adopted child

[c]No formal IQ testing performed. The values reported are an estimate by the clinical geneticist in charge of the case

[d]The mother did not carry the mutation. The father could not be tested

Table S4 (provided as Excel file). Detailed clinical data for the 10 previously described patients carrying *NSD2* variants as well as small deletions encompassing only *NSD2*.

Footnotes to Table S4:

All cDNA and protein positions refer to the NM_133330.2 *NSD2* transcript

L: length; W: weight; H: height; BMI: body mass index; OFC: occipitofrontal circumference; GW: gestational week; ID: intellectual disability; NA: not available; OT: occupational therapy; ST: speech therapy

[a]SDS are given for the 40th gestational week

[b]OFC at 15m: 45,5 cm (-1.98)

Supplemental Materials and Methods

For a list of the reagents used in this study, please refer to Table S2.

Cell Lines

293T (female, embryonic kidney) cells were grown in DMEM medium supplemented with 10% fetal calf serum. HT1080 (human fibrosarcoma) cells were cultured in DMEM medium supplemented with 10% fetal bovine serum and 100 U/mL penicillin/streptomycin. All cells were cultured at 37°C in a humidified incubator with 5% CO_2. Cell lines were authenticated by short tandem repeat profiling and tested negative for mycoplasma (DDC Medical).

Transfection and Viral Transduction

Transient expression was performed using TransIT-293 (Mirus Bio) following the manufacturer's protocol. For NSD2 reconstitution, virus particles were produced by co-transfection of 293T cells with the plvxn-neo-FLAG expressing human NSD2 mutants, pCMV-VSV-G and pCMV-dR8.2 in a ratio of 5:2:3 by mass. 48 hours after transfection, target cells were transduced with 0.45 µm filtered viral supernatant and 8 µg/mL polybrene. Cells were selected 24h after media replacement with 400 µg/mL neomycin after one week selection, the cells expressing human NSD2 were transduced with the lentiCRISPR v2 (with puromycin selection) construct expressing sgControl and sgNSD2. Cells were selected 24h after media replacement with 2 µg/mL puromycin. After 5 days selection, cells were harvested for western blot.

Plasmids and GST fusion protein purification

For bacterial expression, human NSD2 mutants (aa 959-1365) were cloned into pGEX-6P-1. In order to obtain purified GST fusion proteins, these were first expressed in BL21 E.coli by overnight culture at 20°C in LB medium (10 g/L tryptone, 5 g/L yeast extract, and 10 g/L NaCl) supplemented with 0.1 mM IPTG, purified using Glutathione Sepharose 4B and eluted in 10 mM reduced glutathione. Purified proteins were snap frozen and stored in -80°C or used in *in vitro* reaction assays directly. Protein concentrations were measured using Coomassie assay.

In vitro methylation assays

In vitro methylation assays were performed by combining 2 µg recombinant NSD2 mutants and 1 µg substrates (recombinant nucleosomes) in a methyltransferase buffer (50 mM Tris pH 8.0, 20 mM KCl, 5 mM $MgCl_2$, and 10% glycerol) supplemented with 0.1 mM S-adenosylmethionine (SAM). The reaction mixtures were incubated for 2 hours at 30°C. Reactions were analyzed by SDS-PAGE, followed by autoradiography, Coomassie stain or western blot.

Immunoblot analysis

For western blot analysis, cells were lysed in RIPA buffer with 1 mM PMSF and protease inhibitor cocktail. Protein concentration was determined using the Pierce Coomassie Plus Assay. Protein samples were resolved by SDS-PAGE and transferred to a PVDF membrane (0.45 µm). The following antibodies were used (at the indicated dilutions): Tubulin (1:2,000), H3 (1:5,000), H3K36me2 (1:5,000), NSD2 (1:1,000). Secondary antibodies were used at 1:5,000 or 1:10,000 dilution. Protein bands were visualized using ECL detection reagent.
